# Prospective randomised controlled trial comparing PLLA and PLDLA/HA/β‐TCP interference screws in anterior cruciate ligament reconstruction: CT scans, MRI and clinical outcomes

**DOI:** 10.1002/ksa.70024

**Published:** 2025-09-09

**Authors:** Christian Coppola, Raul Mayr, Rene El Attal, Vinzenz Smekal, Gerald Degenhart, Christof Kranewitter, Josef Fritz, Christian Koidl

**Affiliations:** ^1^ Department of Orthopaedics and Trauma Surgery Medical University of Innsbruck Innsbruck Austria; ^2^ Department of Orthopedic Surgery and Traumatology, Hospital of Vipiteno (SABES‐ASDAA) Teaching Hospital of Paracelsus Medical University Vipiteno‐Sterzing Italy; ^3^ Department of Orthopaedics and Trauma Surgery Feldkirch Hospital Feldkirch Austria; ^4^ Department of Orthopaedics and Trauma Surgery AUVA Klagenfurt Klagenfurt Austria; ^5^ Core Facility Micro‐CT, University Clinic for Radiology Medical University of Innsbruck Innsbruck Austria; ^6^ Department of Radiology St. Johann in Tirol Hospital Tirol Austria; ^7^ Institute of Clinical Epidemiology, Public Health, Health Economics, Medical Statistics, and Informatics Medical University of Innsbruck Innsbruck Austria

**Keywords:** anterior cruciate ligament reconstruction, clinical outcome, interference screw, screw resorption, tunnel widening

## Abstract

**Purpose:**

Modifying interference screw composition may ensure better osteoconductive properties in order to reduce tunnel enlargement after anterior cruciate ligament (ACL) reconstruction. The primary and secondary purposes were to evaluate tunnel and screw volume changes in poly‐l‐lactide acid (PLLA) and poly‐d‐lactic acid + hydroxyapatite + β‐tricalcium phosphate (PLDLA+) screws. The tertiary purpose was to compare patient reported‐ and functional outcomes between PLLA and PLDLA+ group. It was hypothesised that PLLA group would show more tunnel enlargement and a lower rate of resorption than PLDLA+ group with similar clinical results.

**Methods:**

Patients were treated with ACL reconstruction using hamstring autograft with femoral and tibial interference screw fixation (PLLA or PLDLA+). Tunnel volume changes were assessed after 0, 24 and 36 months on computed tomography (CT) scans. Screw volume changes were analysed after 0, 3, 6, 12, 24 and 36 months on magnetic resonance imaging (MRI). Patient reported outcome scores (PROMS) and knee laxity were analysed after 12 and 36 months. Data were evaluated using analysis of variance (ANOVA) with Bonferroni correction. Significance was set at <0.05.

**Results:**

Femoral/tibial tunnel volume enlarged 10.7 ± 46.6%/3.8 ± 14.4% (PLLA, *n* = 9) and 2.6 ± 30.8%/19.0 ± 20.5% (PLDLA +, *n* = 13) after 36 months on CT scans (*p* = 0.063/*p* = 0.070). Using MRI scans, femoral/tibial screw volume decreased −53.8 ± 6.9%/−48.8 ± 9% (PLLA) and −88.2 ± 8.9%/−80.6 ± 3.4% (PLDLA+) (*p* ≤ 0.001/*p* ≤ 0.001). No difference was found between the two groups (PLLA, *n* = 17; PLDLA+, *n* = 19) in PROMS and knee laxity (n.s. and/or minimal clinically important difference (MCID) not reached).

**Conclusions:**

Tunnel volumes remained slightly enlarged, screw degradation was significantly higher in the PLDLA+ group while clinical outcomes led to good short‐term results. Comparable tunnel enlargement for both screws can be expected in revision ACL reconstruction.

**Level of Evidence:**

Level I randomised controlled trial.

**Abbreviations**
ACL: anterior cruciate ligament
ANOVA: analysis of variance
CT: computed tomography
HA: hydroxyapatite
IKDC: International Knee Documentation Committee
MRI: magnetic resonance imaging
PLDLA+: poly‐l‐lactide‐*co*‐d‐lactide + HA/β‐TCP
PLLA: poly‐l‐lactic acid
SD: standard deviation
β‐TCP: β‐tricalcium phosphate

## INTRODUCTION

Aperture fixation using interference screws for anterior cruciate ligament (ACL) reconstruction can be performed on both the femoral and tibial sides [33]. It provides ACL graft fixation near to the insertion point [23] and accelerated rehabilitation [14, 30]. In the past, fixation of the ACL graft was widely performed using metallic interference screws [12]. However, the use of metallic screws has declined over the years, since it causes metallic artifacts and graft damage and may complicate revision surgery due to the need for implant removal [1, 12]. Interference screws made of bioabsorbable materials have therefore been increasingly used [20, 26, 31]. Polylactide screws exist in two isomeric forms, l‐lactide and d‐lactide. However, although full material absorption and osteointegration were predicted, even bioabsorbable interference screws have shown disappointing results with regard to bony replacement, tunnel osteolysis, and tunnel enlargement [5, 10, 28]. Pinczewski and Salmon postulated that the cause of this lack of resorption and osteointegration is excess acidic behaviour of the screw material [27]. Adding hydroxyapatite (HA) and β‐tricalcium phosphate (β‐TCP) to polylactide screws buffers the pH decline and provides a scaffold on which new bone can grow [11, 21, 37]. It has been shown in numerous studies that adding HA and β‐TCP to polylactide screws enhances its ability to support new bone formation and resorption of foreign material [2, 4, 6, 21, 33]. Although most clinical studies have not shown any association between tunnel enlargement and the clinical outcome, some studies have reported increases in knee laxity with severely wide ACL tunnels [8, 17, 19, 27, 29]. Excessive tunnel enlargement can complicate revision ACL reconstruction and lead to a need for staged surgery.

The first aim of this prospective randomised controlled trial (RCT) was to evaluate changes in tunnel volumes on computed tomography (CT) between poly‐l‐lactic acid (PLLA) and poly‐l‐lactide‐*co*‐d‐lactide + HA/β‐TCP (PLDLA+) interference screws. The second aim was to evaluate changes in screw volumes on magnetic resonance imaging (MRI) between PLLA and PLDLA+ interference screws. The third aim was to compare patient‐reported outcome measures (PROMS) and knee laxity for between PLLA and PLDLA+ interference screws. It was hypothesised that PLDLA+ interference screw fixation would lead to less tunnel enlargement (first hypothesis) and greater screw degradation (second hypothesis) and would not lead to any difference with regard to PROMS and knee laxity in comparison with PLLA (third hypothesis), 36 months after ACL reconstruction.

## MATERIALS AND METHODS

### Patients

The study was previously approved by the Human Ethics Committee of the Medical University of Innsbruck (Study registration number: UN4185). In this prospectively RCT, patients aged 18–55 years with unilateral ACL ruptures were included for ACL reconstruction with quadrupled hamstrings‐graft using either PLLA or PLDLA+ interference screws. Patients were included if a unilateral ACL rupture had been verified clinically by a positive Lachman test and radiographically by MRI. Exclusion criteria were multiligament injuries, previous ipsilateral knee surgery, cartilage lesions (ICRS grade 2, 3, 4) over 5 cm^2^, concomitant lateral or medial meniscus lesions which were repaired and needed altered rehabilitation protocol, patients which did not have full legal capacity, patients with increased risk of anaesthesia, existing pregnancy and inability to get a CT‐ and/or MRI scan.

### Groups

Two groups were defined in this study in relation to the type of screw used for femoral and tibial graft fixation. In the first group, fixation of the graft was carried out using PLLA interference screws (Arthrex, Inc., Naples, Florida, USA), which are made of poly‐l‐lactic acid. In the second group, fixation of the graft was performed using PLDLA+ interference screws (Arthrex, Inc., Naples, Florida, USA), which are made of 30% biphasic calcium phosphate (HA and ß‐TCP in a 60:40 ratio) and 70% poly‐l‐lactide‐*co*‐d‐lactide (70:30 l:d ratio). Both cannulated screws can be inserted precisely over a guide wire and are introduced using a universal hexalobe drive system. Groups were randomised by using block randomisation. Allocation to one of the two groups was made by opening a sealed envelope.

### Rehabilitation

All patients followed the same supervised physical therapy protocol. A knee brace was used during the first two postoperative weeks, and full weightbearing and a full range of motion were allowed at the third postoperative week. Patients started with closed kinetic chain exercises immediately after surgery. Other noncontact sport activities such as cycling and swimming were allowed 6 weeks after surgery. Running was allowed 3 months postoperatively. Contact or stop‐and‐go sports were allowed after 9 months, depending on the patient's functional stability.

### Radiological measurements

CT scans were performed in 22 patients in the first postoperative week and at 24 and 36 months postoperatively. The interrater correlation coefficient (ICC) for this technique has been shown to be between 0.606 and 0.922 [24, 25]. The CT scans were obtained on a Discovery CT 750 HD (GE Medical Systems, Fairfield, Connecticut, USA). Axial images were performed with coronal and sagittal reconstructions. A single specialist in radiology evaluated the images (K.C.). For volumetric assessment, the postprocessing programme AW2 (GE Medical Systems, Fairfield, Connecticut, USA) was used. Using this programme, tunnels were traced by hand in the axial slices. Slice thickness was set at 0.625 mm (512 × 512 voxels). The sum of all the slices was calculated to obtain tunnel volumes.

MRI scans were performed in 28 patients in the first postoperative week and at 3, 6, 12, 24 and 36 months after surgery. The ICC for this technique has been shown to be between 0.656 and 0.920 [25]. MRI was performed with a 1.5‐Tesla MRI scanner (Avanto Siemens, Erlangen, Germany), acquiring axial T1 tse (TE 11 ms, TR 635), SL 3/3.6 mm, coronal TIRM (TE 15, TR 3139 and TI 150 ms), SL 3/3.6 mm, sagittal T2 Dess3D (TE 8.63 TR 25.2), SL 0.7 mm. For scanning of the tibial and femoral tunnels, sagittal/oblique T1 tse (TE 10 ms, TR 512 ms), SL 3/3.6 mm, PD/T2 tse (TE 14/86, TR 3540), and SL 3/3.6 mm were acquired.

**Figure 1:**
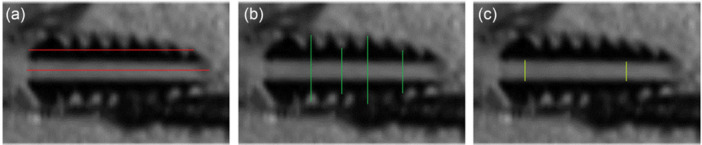
Assessment of the screw volume. (a) Mean length of the screw measured at the centre and the first edge of the screw thread. (b) Mean diameter of the screw measured at four points—twice at the maximum screw thread extension and twice at the minimum screw thread extension. (c) Diameter of the central hole, measured twice.

The postoperative screw volume was checked using microCT (Viva CT 40; Scanco Medical AG, Brüttisellen, Switzerland). The measurement parameters were 70 kV 117 ma 200 ms integration time. Volumes were measured using threshold gauss segmentation and the integrated evaluation software (µCT v6.1; Scanco Medical AG, Brüttisellen, Switzerland). Volumetric assessment was performed using T1 TSE transversal measurements. For proper assessment, the screws had to be aligned longitudinally, using Analyze 12.0 (Analyze Direct Inc., Overland Park, Kansas, USA). The transformed images were then loaded into ImageJ (an open‐source software package), and the measurements shown in Figure 1 were performed. An approximation of the screw volume was calculated on the basis of these measurements using the following formula:

$$V=(\frac{A1+A2}{2}\times {\left(\frac{B1+B2\times B3\times B4}{8}\right)}^{2}-\frac{A1+A2}{2}\times {\left(\frac{C1+C2}{4}\right)}^{2})\times \pi .$$

### Clinical assessments

PROMS and knee laxity were evaluated preoperatively and at 12 and 36 months postoperatively in 36 patients. The (International Knee Documentation Committee) IKDC score and the Lysholm knee score were evaluated for PROMS [15, 18]. The KT1000 arthrometer (Medmetric Corporation, San Diego, California, USA) was used to assess knee laxity. Clinical assessments were performed by a single specialist in orthopaedic surgery (C.K.), which was blinded to the groups.

### Statistical methods

Data for clinical outcomes and for screw and tunnel volumes are presented as means with standard deviation (SD). Changes in tunnel and screw volumes are presented in percentages (%), as means with SD. Parametric tests were carried out, as the analysed data were roughly normally distributed. Differences between groups with regard PROMS, knee laxity, screw and bone tunnel volumes were evaluated using analysis of variance (ANOVA) for repeated measurements, with adjustment for multiple testing using the Bonferroni method. Unpaired *t*‐tests were used to assess for screw and tunnel volume changes in percentages. The chi‐squared test was used to investigate differences in categorical data. Significance was set at <0.05. Statistical analysis was performed using IBM SPSS Statistics for Windows, version 27.0 (IBM Corporation, Armonk, New York, USA).

In three independent datasets comprising between 22 and 36 participants, a Last Observation Carried Forward (LOCF) analysis was performed to simulate the effects of potential loss to follow‐up and assess result stability under conditions of limited sample size. The comparison of group‐level means before and after imputation revealed only minor changes. These results suggest that the overall trends in all datasets are robust and statistically stable, even in the presence of isolated missing data points. This supports the methodological validity of the analyses and highlights the resilience of outcome measures despite small cohort sizes and incomplete follow‐up.

## RESULTS

**Figure 2:**
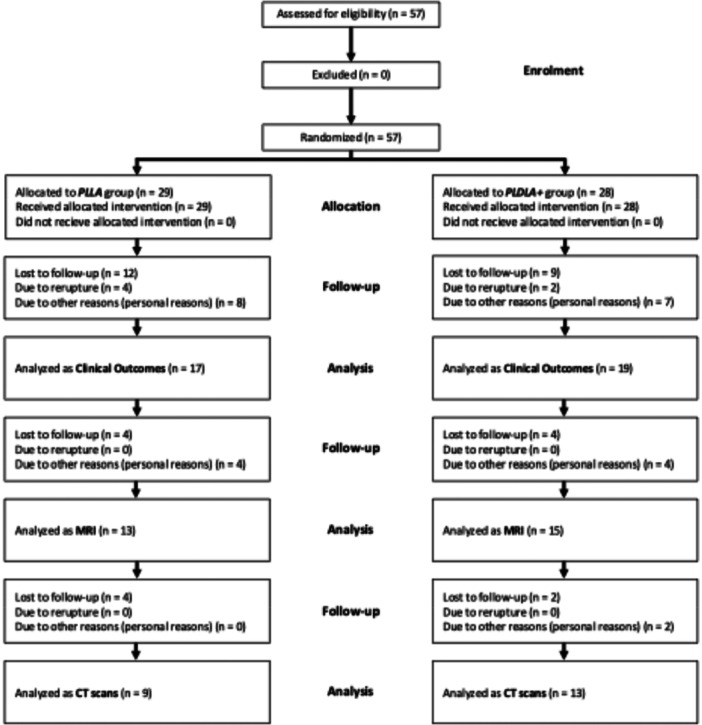
Flow chart showing the progress through the phases of the prospective randomised controlled trial with two parallel groups (enrolment, allocation to intervention, follow‐up and analysis). CT, computed tomography; PLDLA+, poly‐D‐lactic acid + hydroxyapatite + β‐tricalcium phosphate; PLLA, poly‐L‐lactic acid.

A total of 57 patients were included in this RCT. Twenty‐one patients were excluded due to incomplete follow‐up data. Tunnel volumes were evaluated in 22 patients using CT scans immediately after the operation, as well as at 24 and 36 months postoperatively. Screw volumes were measured in 28 patients using MRI scans immediately after the operation, as well as at 3, 6, 12, 24 and 36 months after surgery. PROMS and knee laxity were evaluated for 36 patients preoperatively and at 12 and 36 months postoperatively. Comparison of the baseline characteristics in patients for whom data were available at 36 months and patients with missing information did not show any marked differences, indicating that the potential for selection bias is small. Figure 2 shows the numbers of patients who were evaluated or excluded due to incomplete follow‐up.

### Tunnel volume and tunnel volume changes on CT

**Figure 3:**
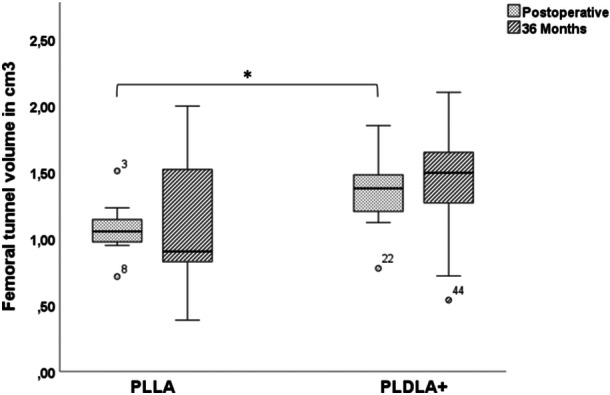
Box plots with medians and interquartile ranges (25%–75%), showing the femoral tunnel volume in cubic centimeters in the two groups on CT scans postoperatively and 36 months after surgery. Significant differences between the groups are marked with an asterisk (*). CT, computed tomography; PLDLA+, poly‐d‐lactic acid + hydroxyapatite + β‐tricalcium phosphate; PLLA, poly‐l‐lactic acid

**Figure 4:**
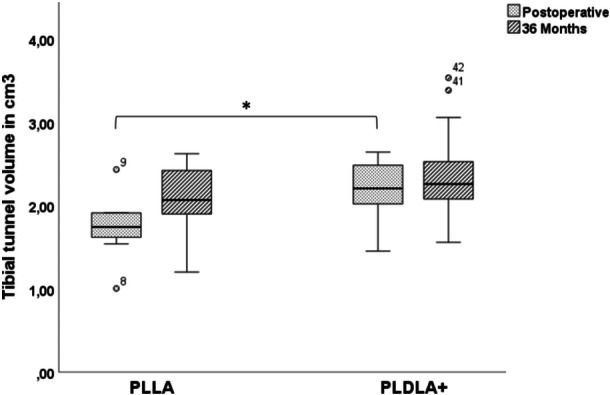
Box plots with medians and interquartile ranges (25%–75%), showing the tibial tunnel volume in cubic centimeters in the two groups on CT scans postoperatively and 36 months after surgery. Significant differences between the groups are marked with an asterisk (*). CT, computed tomography; PLDLA+, poly‐d‐lactic acid + hydroxyapatite + β‐tricalcium phosphate; PLLA, poly‐l‐lactic acid.

Thirty‐six months after operation, the PLLA group showed tunnel volume enlargement by 10.7 ± 46.6% and the PLDLA+ group showed enlargement by 2.6 ± 30.8% on the femoral side (*p* = 0.626). On the tibial side, the PLLA group showed tunnel enlargement by 3.8 ± 14.4% and the PLDLA+ group showed enlargement by 19.0 ± 20.5% (*p* = 0.070). Tables 1, 2, 3 and Figures 3 and 4 present a detailed summary of the findings related to tunnel volumes measurement.

**Table 1:** Demographic data for measurements of tunnel volume and volume changes with CT, showing means and standard deviation for age and sex, classified by group.

	PLLA (*n* = 9)	PLDLA+ (*n* = 13)
Age (years)	30.3 ± 10.4	38 ± 10.6
Sex		
Female	2	3
Male	7	10

Abbreviations: CT, computed tomography; PLDLA+, poly‐d‐lactic acid + hydroxyapatite + β‐tricalcium phosphate; PLLA, poly‐l‐lactic acid.

**Table 2:** Femoral tunnel volume and tunnel volume changes.

Group	Drilled tunnel (mm)	Femoral tunnel (cm^3^)			Change (%)	
		Postoperative	24 months	36 months	24 months	36 months
PLLA (*n* = 9)	6.4 ± 0.5	1.1 ± 0.2	1.4 ± 0.4	1.1 ± 0.5	39.2 ± 51.0	10.7 ± 46.6
PLDLA+ (*n* = 13)	6.5 ± 0.5	1.4 ± 0.3	1.8 ± 0.6	1.4 ± 0.5	23.3 ± 33.8	2.6 ± 30.8
*p*‐value	0.682	0.018	0.115	0.208	0.515	0.626

*Note*: The data are shown as means with standard deviation. Differences between groups for different time points were evaluated using repeated‐measures ANOVA. Differences in femoral tunnel changes were evaluated with an unpaired *t*‐test.

Abbreviations: ANOVA, analysis of variance; PLDLA+, poly‐d‐lactic acid + hydroxyapatite + β‐tricalcium phosphate; PLLA, poly‐l‐lactic acid.

**Table 3:** Tibial tunnel volume and tunnel volume changes.

Group	Drilled tunnel (mm)	Tibial tunnel in cm^3^			Change in%	
		Postoperative	24 months	36 months	24 months	36 months
PLLA (*n* = 9)	8.4 ± 0.7	1.8 ± 0.4	2.1 ± 0.4	2.0 ± 0.5	9.9 ± 12.1	3.8 ± 14.4
PLDLA+ (*n* = 13)	8.8 ± 0.4	2.2 ± 0.3	2.6 ± 0.6	2.4 ± 0.6	24.0 ± 21.3	19.0 ± 20.5
*p*‐value	0.205	0.010	0.029	0.082	0.063	0.070

*Note*: The data are shown as means with standard deviation. Differences between the groups for different time points were evaluated using repeated‐measures ANOVA. Differences in tibial tunnel changes were evaluated with an unpaired *t*‐test.

Abbreviations: ANOVA, analysis of variance; PLDLA+, poly‐d‐lactic acid + hydroxyapatite + β‐tricalcium phosphate; PLLA, poly‐l‐lactic acid.

### Screw volume and screw volume changes on MRI

**Figure 5:**
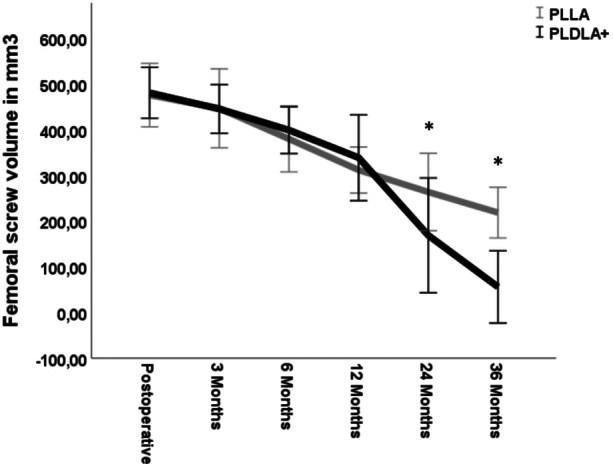
Line diagram with means and standard deviation, comparing the femoral screw volume in cubic millimetres between the two groups on MRI scans at different time points. Significant differences between the groups are marked with an asterisk (*). MRI, magnetic resonance imaging; PLDLA+, poly‐d‐lactic acid + hydroxyapatite + β‐tricalcium phosphate; PLLA, poly‐l‐lactic acid.

**Figure 6:**
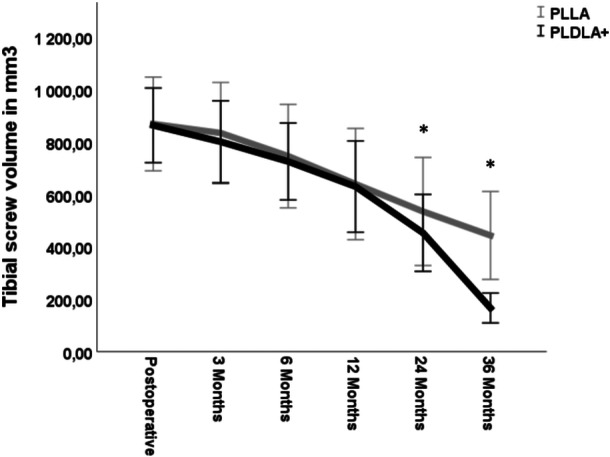
Line diagram with means and standard deviation, comparing the tibial screw volume in cubic millimetres between the two groups on MRI scans at different time points. Significant differences between the groups are marked with an asterisk (*). MRI, magnetic resonance imaging; PLDLA+, poly‐d‐lactic acid + hydroxyapatite + β‐tricalcium phosphate; PLLA, poly‐l‐lactic acid.

The screw volume decreased continuously in both groups from the first postoperative week up to 36 months postoperatively. Thirty‐six months after operation, the PLLA group showed a decrease of –53.8 ± 6.9% and the PLDLA+ group a decrease of –88.2 ± 8.9% of the screw volume on the femoral side (*p* ≤ 0.001). On the tibial side, the PLLA group showed a decrease of –48.8 ± 9.0% and the PLDLA+ group a decrease of –80.6 ± 3.4% of the screw volume (*p* ≤ 0.001). The findings related to screw volumes measurement are summarised in Tables 4, 5, 6 and Figures 5 and 6.

**Table 4:** Demographic data for measurements of screw volume and volume changes with MRI, showing means and standard deviation for age and sex, classified by group.

	PLLA (*n* = 13)	PLDLA+ (*n* = 15)
Age (years)	34.3 ± 11.5	38.1 ± 10.2
Sex		
Female	5	10
Male	8	5

Abbreviations: MRI, magnetic resonance imaging; PLDLA+, poly‐d‐lactic acid + hydroxyapatite + β‐tricalcium phosphate; PLLA, poly‐l‐lactic acid.

**Table 5:** Femoral screw volume and screw volume changes.

Group	Femoral screw volume in mm^3^						Change (%)
	Postoperative	3 months	6 months	12 months	24 months	36 months	0–36 months
PLLA (*n* = 13)	476.0 ± 34.7	447.1 ± 43.2	380.1 ± 36.1	312.1 ± 25.2	263.9 ± 42.4	218.9 ± 27.8	–53.8 ± 6.9
PLDLA+ (*n* = 15)	481.1 ± 27.8	445.7 ± 26.7	399.4 ± 25.6	339.1 ± 47.0	169.1 ± 62.8	56.3 ± 39.6	–88.2 ± 8.9
*p*‐value	0.672	0.917	0.111	0.076	<0.001	<0.001	<0.001

*Note*: The data are shown as means with standard deviation. Differences between the groups for different time points were evaluated using repeated‐measures ANOVA. Differences in screw volume changes were evaluated with an unpaired *t*‐test.

Abbreviations: ANOVA, analysis of variance; PLDLA+, poly‐d‐lactic acid + hydroxyapatite + β‐tricalcium phosphate; PLLA, poly‐l‐lactic acid.

**Table 6:** Tibial screw volume and screw volume changes.

Group	Tibial screw volume mm^3^						Change (%)
	Postoperative	3 months	6 months	12 months	24 months	36 months	0–36 months
PLLA (*n* = 13)	869.1 ± 89.5	834.5 ± 96.5	746.5 ± 98.8	639.7 ± 106.2	535.1 ± 103.1	443.6 ± 83.8	–48.8 ± 9.0
PLDLA+ (*n* = 15)	863.9 ± 71.1	801.2 ± 78.3	725.9 ± 73.3	630.1 ± 87.1	453.3 ± 73.4	166.7 ± 28.6	–80.6 ± 3.4
*p*‐value	0.867	0.322	0.534	0.796	0.022	<0.001	<0.001

*Note*: The data are shown as means with standard deviation. Differences between the groups for different time points were evaluated using repeated‐measures ANOVA. Differences in screw volume changes were evaluated with an unpaired *t*‐test.

Abbreviations: ANOVA, analysis of variance; PLDLA+, poly‐d‐lactic acid + hydroxyapatite + β‐tricalcium phosphate; PLLA, poly‐l‐lactic acid.

### PROMS and knee laxity

Twelve months postoperatively, the IKDC in the PLLA group was 82.0 ± 11.1, in comparison with 89.1 ± 9.5 in the PLDLA+ group (*p* = 0.049). Thirty‐six months postoperatively, the IKDC in the PLLA group was 89.9 ± 9.5, in comparison with 95.6 ± 6.4 in the PLDLA+ group (*p* = 0.042). The mean change did not reach the established minimal clinically important difference (MCID) of 11.5 points [16], suggesting limited clinical relevance. There were no significant differences in the Lysholm score nor clinical relevance (MCID not exceeded) was observed between the two groups at 12 and 36 months postoperatively [9]. There were no significant differences in knee laxity measured with the arthrometer between the two groups at 12 and 36 months postoperatively. Tables 7, 8, 9, 10 present a detailed summary of the findings related to clinical outcomes.

**Table 7:** Demographic data for the evaluation of clinical outcomes, showing means and standard deviation for age and sex, sorted by group.

	PLLA (*n* = 17)	PLDLA+ (*n* = 19)
Age (years)	33.0 ± 11.2	35.7 ± 10.9
Sex		
Female	3	6
Male	14	13

Abbreviations: PLDLA+, poly‐d‐lactic acid + hydroxyapatite + β‐tricalcium phosphate; PLLA, poly‐l‐lactic acid.

**Table 8:** Means and standard deviation for International Knee Documentation Committee (IKDC) scores preoperatively and at 12 and 36 months postoperatively.

Group	Preoperative	12 months	36 months	Preoperative vs. 36 months
PLLA (*n* = 17)	62.7 ± 14.7	82.0 ± 11.1	89.9 ± 9.5	*p* ≤ 0.001
PLDLA+ (*n* = 19)	66.6 ± 17.1	89.1 ± 9.5	95.6 ± 6.4	*p* ≤ 0.001
*p*‐value		0.049	0.042	

*Note*: Differences between the groups (preoperatively and at 12 months and 36 months postoperatively) and differences across time points (preoperatively and 36 months postoperatively) were evaluated using repeated‐measures ANOVA.

Abbreviations: ANOVA, analysis of variance; PLDLA+, poly‐d‐lactic acid + hydroxyapatite + β‐tricalcium phosphate; PLLA, poly‐l‐lactic acid.

**Table 9:** Means and standard deviation for the Lysholm score preoperatively and at 12 and 36 months postoperatively.

Group	Preoperative	12 months	36 months	Preoperative vs. 36 months
PLLA (*n* = 17)	71.5 ± 13.7	92.7 ± 6.4	96.5 ± 4.6	*p* ≤ 0.001
PLDLA+ (*n* = 19)	66.0 ± 18.1	95.4 ± 4.6	96.2 ± 4.7	*p* ≤ 0.001
*p*‐value		0.157	0.869	

*Note*: Differences between the groups (preoperatively and at 12 months and 36 months postoperatively), as well as differences across time points (preoperatively and 36 months postoperatively) were evaluated using repeated‐measures ANOVA.

Abbreviations: ANOVA, analysis of variance; PLDLA+, poly‐d‐lactic acid + hydroxyapatite + β‐tricalcium phosphate; PLLA, poly‐l‐lactic acid.

**Table 10:** Knee laxity measured with the KT1000 arthrometer preoperatively and at 12 and 36 months postoperatively.

Group	Preoperative	12 months	36 months	Preoperative vs. 36 months
PLLA (*n* = 17)	17 × 6–10 mm	10 × 3–5 mm 7 × 1–2 mm	10 × 3–5 mm 7 × 1–2 mm	*p* ≤ 0.001
PLDLA+ (*n* = 19)	19 × 6–10 mm	7 × 3–5 mm 12 × 1–2 mm	7 × 3–5 mm 12 × 1–2 mm	*p* ≤ 0.001
*p*‐value		0.187	0.187	

*Note*: Differences between categorical data were evaluated using the chi‐squared test.

Abbreviations: PLDLA+, poly‐d‐lactic acid + hydroxyapatite + β‐tricalcium phosphate; PLLA, poly‐l‐lactic acid.

## DISCUSSION

In the present study, tunnel enlargement at 36 months postoperatively was slightly, but not significantly, less in the PLDLA+ group in comparison with the PLLA group (rejecting first hypothesis). Tunnel volumes enlarged similarly in both groups at 24 months after surgery. From 24 to 36 months postoperatively, a reduction in tunnel volumes was observed in both groups, although tunnel volumes remained slightly enlarged. Similar results were reported by Sundaraj et al. [33], which investigated tunnel volumes in PLLA‐HA screws at 2, 5 and 13 years after ACL reconstruction. Tunnel volumes increased from 2 to 5 years after ACL reconstruction. After 13 years, the femoral tunnel volume had declined, whereas the tibial tunnel volume remained slightly enlarged. Delayed bone replacement is a frequently seen phenomenon, which may complicate revision surgery [10, 25, 32, 34]. Composite screws have been introduced to enhance bony integration [3, 27]. A review investigating composite implants reported that osteoconductivity at the implant site was identified in 63% of cases, while tunnel widening was only seen in 3% [7]. Lee et al. reported that the addition of HA to PLLA screws reduced the extent of tibial tunnel widening significantly in comparison with the PLLA group [20]. Paradoxically, tunnel enlargement was also reported using interference screws containing β‐TCP [36]. Tecklenburg et al. did not observe any bony replacement 24 months postoperatively using either PLLA‐HA nor PLLA–β‐TCP screws, despite achieving excellent clinical results [34]. These findings raise doubts about the clinical relevance of tunnel enlargement.

This study showed that there was significantly greater screw degradation in PLDLA+ screws in comparison with the PLLA screws (confirming second hypothesis). After 12 months, screw degradation did not show any significant differences between groups. After that point, a significant difference in screw degradation between groups was observed. Gradual screw degradation was similar to that reported in other studies with bioabsorbable screws [2, 7, 12, 33, 34], although there are also studies reporting delayed and nongradual resorption behaviour [28, 35]. Drogset et al. was not able to detect intact PLLA screws in any patient after a 7‐year follow‐up [12]. Lee et al. reported a resorption rate of 41.3 ± 12.0% 24 months after ACL reconstruction with PLLA screws [20]. Pinczewski et al. reported that the femoral and tibial PLLA‐HA screws were a mean of 76% and 68% of their original volume at 2 years and 36% and 46% at 5 years after ACL reconstruction [2, 33]. Overall, resorption rates observed are in line with those of comparable studies, indicating that screw resorption occurs over a long period. Bioabsorbable screws with the addition of ‘osteoconductive’ materials such as HA or β‐TCP have been the subject of previous investigations, with unclear results [4, 6, 27, 36]. However, the addition of osteoconductive materials appear to provide an advantage in relation to screw degradation.

There were no significant differences in knee laxity and the Lysholm score between groups. The IKDC score was significantly higher in the PLDLA+ group but did not meet the threshold for MCID, suggesting limited clinical relevance (partly confirming third hypothesis). PROMS and knee laxity improved significantly from time 0 to 36 months after surgery, exceeding the respective MCID thresholds, underscoring the clinical relevance of the outcomes. In comparable studies, bioabsorbable screws have similarly been associated with good clinical outcomes in, although tunnel enlargement or cyst formation have been reported [2, 20, 33, 36]. These studies did not observe any differences with regard to clinical outcomes between PLLA and other screw types.

This study presents some limitations. First of all, final sample size is small. Many patients had to be excluded due to loss to follow‐up. However, baseline comparisons between patients who completed the 36‐month follow‐up and those who did not showed no meaningful differences in key demographic or clinical measures. A power analysis was not adopted. However, a secondary analysis in which the last observation carried forward (LOCF‐Analysis) approach was performed. The results suggest that the overall trends in all datasets are robust and statistically stable and highlights the resilience of outcome measures. Although statistical significance was achieved for many end points, a larger sample size would provide greater confidence in interpretating the results. Since long‐term studies have shown prolonged degradation as well as a decrease in tunnel volume over time [6, 7, 33], a longer follow‐up period would be of further interest. In addition, not all patients with clinical outcome data also underwent MRI and CT examinations. The correlation between tunnel enlargement and clinical outcomes is therefore limited. One strength of the study is the combination of CT and MRI, which have been reported to be the most valuable instruments for tunnel and screw investigation after ACL reconstruction [2, 10, 13, 22, 24, 25, 33]. PROMS and knee laxity were assessed in order to evaluate the clinical validity of the results.

## CONCLUSION

In conclusion, the tunnel volume enlarged similarly in the two groups and remained slightly enlarged in comparison with the postoperative measurements. Continuous screw degradation was observed in both groups, with significantly greater degradation in the PLDLA+ group in comparison with the PLLA group 36 months after ACL reconstruction.

## AUTHOR CONTRIBUTIONS

*Study design*: Rene El Attal, Vinzenz Smekal, and Christian Koidl. *Data analysis*: Christian Coppola, Christof Kranewitter, Gerald Degenhart, and Josef Fritz. *Manuscript writing*: Christian Coppola, Raul Mayr, and Christof Kranewitter. *Critically revising, proofreading and commenting*: Christian Coppola, Rene El Attal, Vinzenz Smekal, Raul Mayr, Josef Fritz, and Gerald Degenhart. All authors read and approved the final manuscript.

## CONFLICT OF INTEREST STATEMENT

The authors declare no conflicts of interest.

## ETHICS STATEMENT

Human Ethics Committee of the Medical University of Innsbruck; Study registration number: UN4185. All patients gave written consent for the study.

## Data Availability

Encourages data sharing.

## References

[1] Almazán A , Miguel A , Odor A , Ibarra JC . Intraoperative incidents and complications in primary arthroscopic anterior cruciate ligament reconstruction. Arthroscopy. 2006;22:1211–1217. 17084299 10.1016/j.arthro.2006.06.019

[2] Arama Y , Salmon LJ , Sri‐Ram K , Linklater J , Roe JP , Pinczewski LA . Bioabsorbable versus titanium screws in anterior cruciate ligament reconstruction using hamstring autograft: a prospective, blinded, randomized controlled trial with 5‐year follow‐up. Am J Sports Med. 2015;43:1893–1901. 26109611 10.1177/0363546515588926

[3] Athanasiou K , Agrawal C , Barber F , Burkhart S . Orthopaedic applications for PLA‐PGA biodegradable polymers. Arthroscopy. 1998;14:726–737. 9788368 10.1016/s0749-8063(98)70099-4

[4] Barber FA , Dockery WD . Long‐term absorption of β–tricalcium phosphate poly‐L‐lactic acid interference screws. Arthroscopy. 2008;24:441–447. 18375277 10.1016/j.arthro.2007.10.004

[5] Barber FA , Dockery WD . Long‐term absorption of poly‐L‐lactic acid interference screws. Arthroscopy. 2006;22:820–826. 16904577 10.1016/j.arthro.2006.04.096

[6] Barber FA , Dockery WD , Hrnack SA . Long‐term degradation of a poly‐lactide co‐glycolide/β‐tricalcium phosphate biocomposite interference screw. Arthroscopy. 2011;27:637–643. 21429700 10.1016/j.arthro.2010.11.056

[7] Barber FA , Spenciner DB , Bhattacharyya S , Miller LE . Biocomposite implants composed of poly(lactide‐co‐glycolide)/β‐tricalcium phosphate: systematic review of imaging, complication, and performance outcomes. Arthroscopy. 2017;33:683–689. 27998641 10.1016/j.arthro.2016.09.032

[8] Bourke HE , Salmon LJ , Waller A , Winalski CS , Williams HA , Linklater JM , et al. Randomized controlled trial of osteoconductive fixation screws for anterior cruciate ligament reconstruction: a comparison of the Calaxo and Milagro screws. Arthroscopy. 2013;29:74–82. 23276415 10.1016/j.arthro.2012.10.021

[9] Briggs KK , Lysholm J , Tegner Y , Rodkey WG , Kocher MS , Steadman JR . The reliability, validity, and responsiveness of the Lysholm score and Tegner activity scale for anterior cruciate ligament injuries of the knee: 25 years later. Am J Sports Med. 2009;37:890–897. 19261899 10.1177/0363546508330143

[10] Coppola C , Krost S , Runer A , Raas C , Glodny B , Mayr R . PEEK interference screws show significant tunnel enlargement after ACL reconstruction and is comparable to adjustable‐length loop cortical button fixation. Indian J Orthop. 2024;58:40–47. 38161404 10.1007/s43465-023-01029-8 PMC10754774

[11] Daculsi G , Laboux O , Malard O , Weiss P . Current state of the art of biphasic calcium phosphate bioceramics. J Mater Sci: Mater Med. 2003;14:195–200. 15348464 10.1023/a:1022842404495

[12] Drogset JO , Straume LG , Bjørkmo I , Myhr G . A prospective randomized study of ACL‐reconstructions using bone‐patellar tendon‐bone grafts fixed with bioabsorbable or metal interference screws. Knee Surg Sports Traumatol Arthrosc. 2011;19:753–759. 21234545 10.1007/s00167-010-1353-4 PMC3076560

[13] Foldager C , Jakobsen BW , Lund B , Christiansen SE , Kashi L , Mikkelsen LR , et al. Tibial tunnel widening after bioresorbable poly‐lactide calcium carbonate interference screw usage in ACL reconstruction. Knee Surg Sports Traumatol Arthrosc. 2010;18:79–84. 19609505 10.1007/s00167-009-0865-2

[14] Harvey A , Thomas NP , Amis AA . Fixation of the graft in reconstruction of the anterior cruciate ligament. J Bone Joint Surg Br. 2005;87:593–603. 15855357 10.1302/0301-620X.87B5.15803

[15] Irrgang JJ , Anderson AF , Boland AL , Harner CD , Kurosaka M , Neyret P , et al. Development and validation of the international knee documentation committee subjective knee form. Am J Sports Med. 2001;29:600–613. 11573919 10.1177/03635465010290051301

[16] Irrgang JJ , Anderson AF , Boland AL , Harner CD , Neyret P , Richmond JC , et al. Responsiveness of the International Knee Documentation Committee Subjective Knee Form. Am J Sports Med. 2006;34:1567–1573. 16870824 10.1177/0363546506288855

[17] Kaeding C , Farr J , Kavanaugh T , Pedroza A . A prospective randomized comparison of bioabsorbable and titanium anterior cruciate ligament interference screws. Arthroscopy. 2005;21:147–151. 15689862 10.1016/j.arthro.2004.09.012

[18] Kocher MS , Steadman RJ , Briggs KK , Sterett WI , Hawkins RJ . Reliability, validity, and responsiveness of the Lysholm knee scale for various chondral disorders of the knee. J Bone Joint Surg‐Am Vol. 2004;86:1139–1145. 10.2106/00004623-200406000-00004 15173285

[19] Kousa P , Järvinen TLN , Kannus P , Järvinen M . Initial fixation strength of bioabsorbable and titanium interference screws in anterior cruciate ligament reconstruction. Biomechanical evaluation by single cycle and cyclic loading. Am J Sports Med. 2001;29:420–425. 11476379 10.1177/03635465010290040601

[20] Lee DW , Lee JW , Kim SB , Park JH , Chung KS , Ha JK , et al. Comparison of poly‐L‐lactic acid and poly‐L‐lactic acid/hydroxyapatite bioabsorbable screws for tibial fixation in ACL reconstruction: clinical and magnetic resonance imaging results. Clin Orthop Surg. 2017;9:270–279. 28861193 10.4055/cios.2017.9.3.270 PMC5567021

[21] Macarini L , Milillo P , Mocci A , Vinci R , Ettorre GC . Poly‐L‐lactic acid‐hydroxyapatite (PLLA‐HA) bioabsorbable interference screws for tibial graft fixation in anterior cruciate ligament (ACL) reconstruction surgery: MR evaluation of osteointegration and degradation features. Radiol Med. 2008;113:1185–1197. 18956150 10.1007/s11547-008-0334-x

[22] Marchant MH , Willimon SC , Vinson E , Pietrobon R , Garrett WE , Higgins LD . Comparison of plain radiography, computed tomography, and magnetic resonance imaging in the evaluation of bone tunnel widening after anterior cruciate ligament reconstruction. Knee Surg Sports Traumatol Arthrosc. 2010;18:1059–1064. 19953224 10.1007/s00167-009-0952-4

[23] Mayr R , Heinrichs CH , Eichinger M , Coppola C , Schmoelz W , Attal R . Biomechanical comparison of 2 anterior cruciate ligament graft preparation techniques for tibial fixation: adjustable‐length loop cortical button or interference screw. Am J Sports Med. 2015;43:1380–1385. 25767269 10.1177/0363546515574062

[24] Mayr R , Smekal V , Koidl C , Coppola C , Eichinger M , Rudisch A , et al. ACL reconstruction with adjustable‐length loop cortical button fixation results in less tibial tunnel widening compared with interference screw fixation. Knee Surg Sports Traumatol Arthrosc. 2020;28:1036–1044. 31372680 10.1007/s00167-019-05642-9

[25] Mayr R , Smekal V , Koidl C , Coppola C , Fritz J , Rudisch A , et al. Tunnel widening after ACL reconstruction with aperture screw fixation or all‐inside reconstruction with suspensory cortical button fixation. Knee. 2017;24:1047–1054. 28705571 10.1016/j.knee.2017.06.007

[26] McDermott ER , Aman ZS , Dekker TJ . Anterior cruciate ligament reconstruction: fixation techniques. Arthroscopy. 2024;40:201–203. 38296430 10.1016/j.arthro.2023.11.005

[27] Pinczewski LA , Salmon LJ . Editorial commentary: the acrid bioscrew in anterior cruciate ligament reconstruction of the knee. Arthroscopy. 2017;33:2195–2197. 29198356 10.1016/j.arthro.2017.08.229

[28] Radford MJ , Noakes J , Read J , Wood DG . The natural history of a bioabsorbable interference screw used for anterior cruciate ligament reconstruction with a 4‐strand hamstring technique. Arthroscopy. 2005;21:707–710. 15944627 10.1016/j.arthro.2005.03.005

[29] Saccomanno MF , Shin JJ , Mascarenhas R , Haro M , Verma NN , Cole BJ , et al. Clinical and functional outcomes after anterior cruciate ligament reconstruction using cortical button fixation versus transfemoral suspensory fixation: a systematic review of randomized controlled trials. Arthroscopy. 2014;30:1491–1498. 25064753 10.1016/j.arthro.2014.05.028

[30] Shelbourne KD , Nitz P . Accelerated rehabilitation after anterior cruciate ligament reconstruction. Am J Sports Med. 1990;18:292–299. 2372081 10.1177/036354659001800313

[31] Shumborski S , Heath E , Salmon LJ , Roe JP , Linklater JP , Facek M , et al. A Randomized controlled trial of PEEK versus titanium interference screws for anterior cruciate ligament reconstruction with 2‐year follow‐up. Am J Sports Med. 2019;47:2386–2393. 31306589 10.1177/0363546519861530

[32] Stener S , Ejerhed L , Sernert N , Laxdal G , Rostgård‐Christensen L , Kartus J . A long‐term, prospective, randomized study comparing biodegradable and metal interference screws in anterior cruciate ligament reconstruction surgery: radiographic results and clinical outcome. Am J Sports Med. 2010;38:1598–1605. 20392970 10.1177/0363546510361952

[33] Sundaraj K , Salmon LJ , Heath EL , Winalski CS , Colak C , Vasanji A , et al. Bioabsorbable versus titanium screws in anterior cruciate ligament reconstruction using hamstring autograft: a prospective, randomized controlled trial with 13‐year follow‐up. Am J Sports Med. 2020;48:1316–1326. 32302205 10.1177/0363546520911024

[34] Tecklenburg K , Burkart P , Hoser C , Rieger M , Fink C . Prospective evaluation of patellar tendon graft fixation in anterior cruciate ligament reconstruction comparing composite bioabsorbable and allograft interference screws. Arthroscopy. 2006;22:993–999. 16952730 10.1016/j.arthro.2006.05.010

[35] Thompson SM , Fung S , Wood DG . The natural history of biointerference screw cyst and new bone formation in anterior cruciate ligament reconstruction: 16‐year follow‐up. Am J Sports Med. 2016;44:113–117. 26473011 10.1177/0363546515608479

[36] Wang JH , Lee ES , Lee BH . Paradoxical tunnel enlargement after ACL reconstruction with hamstring autografts when using β‐TCP containing interference screws for tibial aperture fixation‐ prospectively comparative study. BMC Musculoskelet Disord. 2017;18:398. 28915914 10.1186/s12891-017-1757-0 PMC5602947

[37] Weiler A , Hoffmann RFG , Stähelin AC , Helling HJ , Südkamp NP . Biodegradable implants in sports medicine: the biological base. Arthroscopy. 2000;16:305–321. 10750011 10.1016/s0749-8063(00)90055-0

